# Supplementary material for: Concurrent diffusion of nicotinic acetylcholine receptors and fluorescent cholesterol disclosed by two-colour sub-millisecond MINFLUX-based single-molecule tracking
Source: Nat Commun. 2025 Jul 9;16:6336. doi: 10.1038/s41467-025-61489-4 (PMC12241495; doi:10.1038/s41467-025-61489-4)
Supplement: Supplementary file 1 — Supplementary Information Fixed 23.06 [file 41467_2025_61489_MOESM1_ESM.pdf]

Concurrent diffusion of nicotinic acetylcholine receptors and fluorescent cholesterol disclosed by two-colour sub-millisecond MINFLUX-based single-molecule tracking

Francesco Reina, Lucas A. Saavedra, Christian Eggeling and Francisco J. Barrantes

Supplementary Material

Nicotinic acetylcholine receptor topography at the surface of CHO-K1/A5 cells.

The clonal cell line CHO-K1/A5 (Roccamo et al. 1999) expresses robust amounts of nicotinic acetylcholine receptor (nAChR) at its cell surface. This is the cell line used in this work to study the translational dynamics of the nAChR and the fluorescent cholesterol analogue fPEG-Chol (Sato et al. 2004; Kamerbeek et al. 2013). Cells were incubated on ice with either CF®640R-BTX, CF®680R-BTX, fPEG-Chol or a combination of CF®680R-BTX + fPEG-Chol in 1 mL of culture medium. After several initial assays the final concentration of the two fluorescent α-bungarotoxin derivatives was kept at 500 pM, and that of the fluorescent cholesterol analogue at 1 nM. At these concentrations, only a fraction of the nAChRs were labelled, thus meeting the conditions for MINFLUX single-molecule tracking (SMT). These very low dye concentrations employed for the MINFLUX experiments are not adequate for imaging purposes; concentrations three orders of magnitude higher were required for wide-field imaging of fPEG-Chol (see Supplementary Figure in ref. (Kamerbeek et al. 2013)). Labelling conditions to delineate the distribution of nAChRs requires label concentrations in the micromolar range (Supplementary Figure 1)

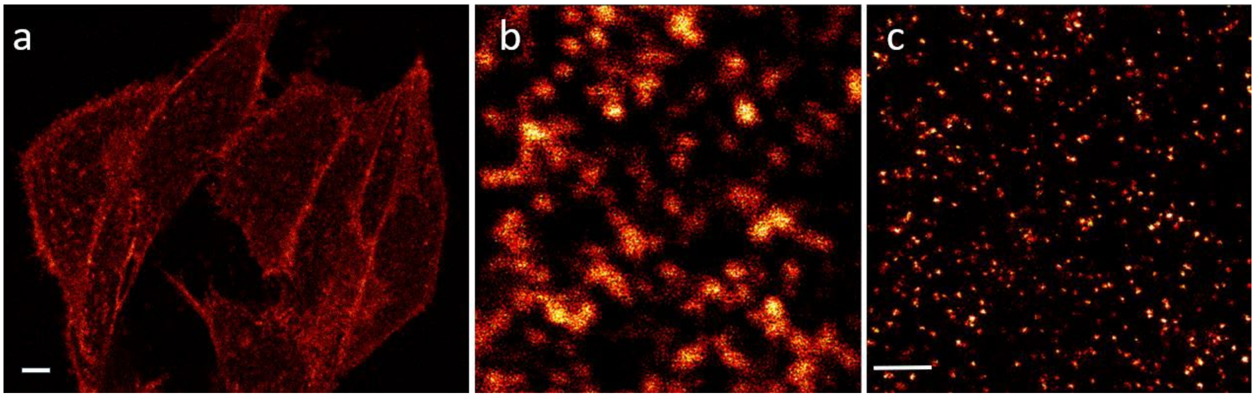

Supplementary Figure 1. nAChR imaged in confocal and STED modes at micromolar CF®680R-BTX concentrations. a) CHO-K1/A5 cells labelled with CF®680R-BTX at 4℃ for 20 min and imaged in the confocal model upon excitation at 640 nm at an emission wavelength between 660 and 750 nm using an InfinityLine Abberior STED microscope. The coverglass-contacting ventral surface of the cells was focused using a 1.4 N.A. oil-immersion objective and a pixel size of 50 nm. b) Confocal image of a ROI of a CHO-K1/A5 cell stained with Alexa-Fluor 647 using an Abberior Stedycon STED microscope and a 1.4 N.A. objective. c) STED superresolution image of (b). Scale bar in (a) is 10 µm; the bar shown in (c) applies to (b) and (c) and corresponds to 1 µm.

Detector Channel Ratio (DCR): discrimination of toxin and cholesterol probe signals

As indicated in the main text, dissection of the trajectories of the nAChR macromolecule and the cholesterol analogue relied on the ratiometric dissection of their emission signals upon excitation with a single laser wavelength in the MINFLUX setup. DCR is calculated as $I_1/(I_1+I_2)$ such that I_1 and I_2 are the intensity obtained by both detectors used in MINFLUX to make a localization. A dye which emits only in I_1 will have a DCR value of 1. However, if intensity is obtained only through I_2, the DCR of the same dye will be 0. Supplementary Figure 2 schematically depicts the DCR as the average DCR value in a single trajectory and its relationship with the Effective Frequency at Offset (EFO) which refers to the average frequency in Hz of the photons collected at the outer points of the MINFLUX pattern. The difference of DCR distribution between CF®680R-BTX and fPEG-Chol is apparent ($p<0.001$), justifying the use of the ratiometric approach to discriminate between the two probes in 2-colour experiments.

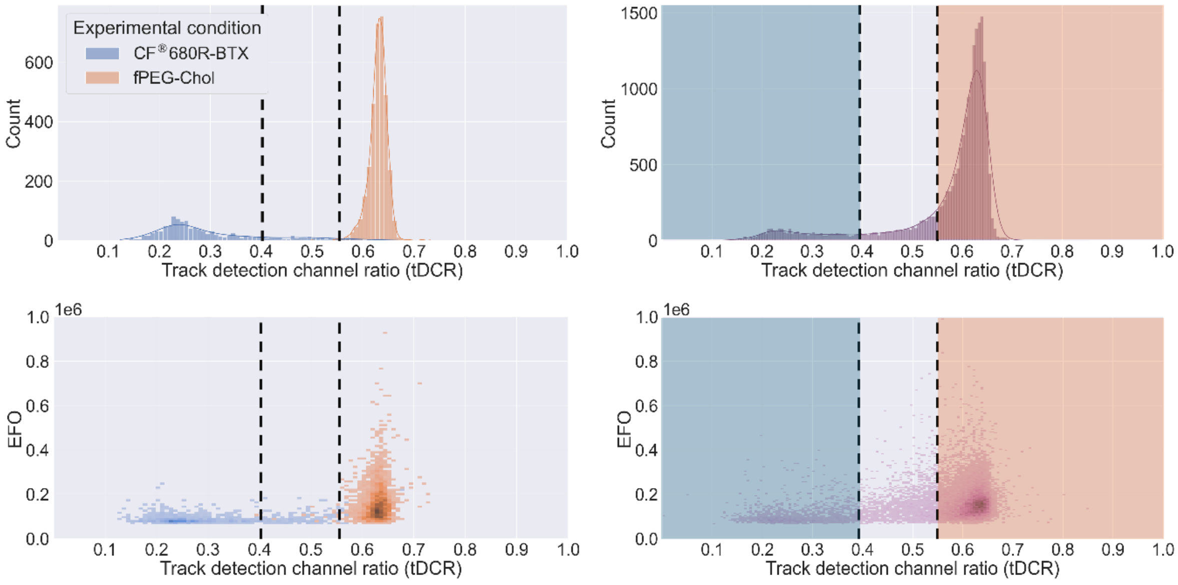

Supplementary Figure 2. DCR and EFO analysis. DCR and DCR vs EFO histograms of experiments with CF®680R-BTX and fPEG-Chol separately (*left*) and the same in experiments with simultaneous staining with CF®680R-BTX and fPEG-Chol (*right*). Notice the difference between the two mean distributions. The right vertical dashed line indicates the manually selected DCR threshold (=0.55) to isolate trajectories with fPEG-Chol and BTX680R when both experimental labelling conditions were sampled simultaneously. When fluorescent-labelled nAChR and fPEG-Chol are co-tracked the interaction between the receptor macromolecules and the fluorescent cholesterol can be detected between the left (0.40) and right (0.55) dashed lines, as shown in the highlighted central area.

Initial characterisation of nAChR and fluorescent cholesterol trajectories

Trajectories were first characterized in terms of extent and duration of the tracks and number of steps recorded in MINFLUX. Similar elementary parameters were quantified for the individual steps, as listed in Supplementary Table 1 below.

Supplementary Table 1*. SMT elementary parameters of nAChR and fluorescent cholesterol under different experimental conditions

Parameter	Mean ± S.E.M			
	L' [number of steps]	T [track duration, s]	$\Delta_{t_i'}$[step duration, μs]	$\Delta_{x_i'}$[displacement, nm]
CF®640R-BTX-labeled nAChR (n=3,006)*	1105 ± 26	0.948 ± 0.022	858.68 ± 0.61	15 ± 1
CF®640R-BTX-labeled nAChR + CDx (n=2,474)	793 ± 27	0.724 ± 0.019	914.34 ± 0.87	15 ± 1
Cholesterol analogue (fPEG-Chol) alone (n=1,478)	1012 ± 31	0.497 ± 0.017	492.18 ± 0.63	14 ± 1
Far-red toxin (CF®680R-BTX) alone (n=404)	925 ± 63	0.989 ± 0.063	1069.76 ± 2.32	15 ± 1
fPEG-Chol in the presence of far-red (CF®680R-BTX) toxin (n=7,057)	1496 ± 23	0.508 ± 0.008	340.01 ± 0.15	16 ± 1
Far-red toxin (CF®680R-BTX) in the presence of fPEG-Chol (n=2,219)	2034 ± 62	1.076 ± 0.030	529.29 ± 0.15	14 ± 1
CF®640R-BTX + 50 nM cholesterol (n=1,299)	972 ± 32	0.664 ± 0.022	684.75 ± 0.41	14 ± 1
CF®640R-BTX (n=734; low density) + 50 nM cholesterol	453 ± 21	0.948 ± 0.022	689.06 ± 1.35	17 ± 1
fPEG-Chol in the presence of far-red toxin (CF®680R-BTX) + 50 nM cholesterol (n=1,007)	816 ± 34	0.346 ± 0.015	424.50 ± 0.70	17 ± 1
Far-red toxin (CF®680R-BTX) in the presence of fPEG-Chol + 50 nM cholesterol (n=146)	435 ± 54	0.389 ± 0.018	895.44 ± 5.71	15 ± 1
fPEG-Chol in the presence of far-red (CF®680R-BTX) + 100 nM cholesterol (n=1,119)	1316 ± 48	0.467 ± 0.018	355.43 ± 0.41	16 ± 1
Far-red toxin (CF®680R-BTX) in the presence of fPEG-Chol + 100 nM cholesterol (n=683)	1213 ± 55	0.702 ± 0.029	579.19 ± 1.06	15 ± 1

*n indicates the number of trajectories

Immobile particle exclusion

Supplementary Figure 3 shows the distribution of mobile/immobile trajectories for the different experimental conditions tested upon application of the criterion of ref. (Golan and Sherman 2017). About ~52%, ~63%, ~52%, of the trajectories from CF®640R-BTX, cholesterol-depleted ("CDx"), and CF®680R-BTX-only samples were classified as immobile, respectively. In contrast, only ~20% immobile tracks were observed for trajectories from fPEG-Chol samples either co-labelled or not with CF®680R-BTX. The number of steps of the immobile trajectories were less than 10% of those of mobile trajectories.

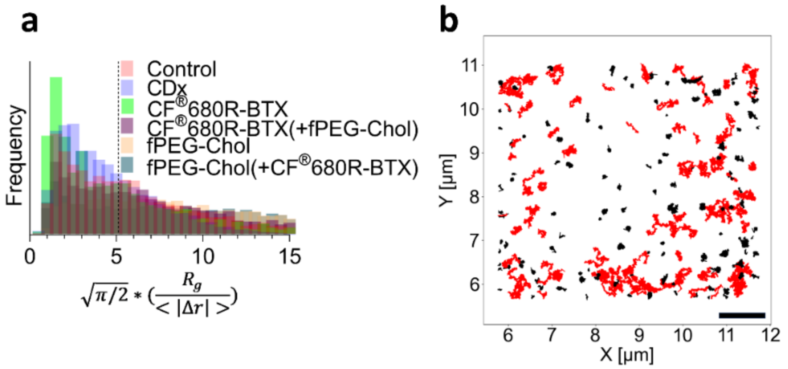

Supplementary Figure 3. Classification of trajectories into mobile and immobile (Golan and Sherman 2017). a) Distribution of the ratio of the radius of gyration and the mean step size of the recorded trajectories. Ratios between 5.121 and 5.300 (95% confidence) were obtained and a cutoff value of 5.121 was chosen (dashed vertical line). b) A region of interest (ROI) showing trajectories from control cells labelled with CF®640R-BTX upon classification into immobile (black) and mobile (red) populations. Scale bar = 1 µm.

Extraction of kinetic parameters from MSD curves

As shown in Figure 1 and Table 1 in the main text, MSD analysis rendered kinetic parameters related to the diffusion regimes followed by the nAChR labelled with the far-red toxin and the fluorescent cholesterol probe. Supplementary Table 2 shows the man-tailored effect of cholesterol depletion and exogenous cholesterol addition on these parameters using feeble effect of 50 nM exogenous cholesterol supplementation on toxin-labelled nAChR and fPEG-Chol diffusion. Only at 100 nM cholesterol addition did the diffusion coefficients increase, albeit modestly ($p<0.01$). Supplementary Table 1 also provides the kinetic parameters of the diffusion following perturbation of the actin cytoskeleton, which will be discussed in more detail in a later section.

Supplementary Table 2 summarises the generalized anomalous diffusion coefficient and apparent anomalous coefficient averaged over the entire ensemble population of trajectories and the average time that the trajectories spend in free-walk, non-confined portions and the confinement sojourns, respectively.

Supplementary Table 2*. Effect of cholesterol depletion or exogenous cholesterol addition on the generalized diffusion coefficient (K_β) and apparent anomalous coefficient (β) averaged over all trajectories, duration of confined sojourns and non-confined portions, and their ratio (r)

Experimental condition	Mean ± S.E.M.				
	K_β ($\mu m^2 s^{-\beta}$)	β	Average duration of confinement sojourn [ms]	Average duration of non-confined portion [ms]	#Confined ratio, *r*
**CF®640R-BTX-labelled nAChR**	**0.301 ± 0.023**	**1.09 ± 0.01**	**71 ± 8**	**12 ± 1**	**0.76 ± 0.02**
**CF®640R-BTX-labelled nAChR (+cholesterol depleted via CDx)**	0.189 ± 0.016	1.06 ± 0.02	137 ± 24	15 ± 1	0.75 ± 0.02
**CF®640R-BTX-labelled nAChR + 50 nM cholesterol**	0.517 ± 0.074	1.20 ± 0.05	55 ± 10	12 ± 2	0.72 ± 0.05
**fPEG-Chol in the presence of far-red toxin (CF®680R-BTX) + 50 nM cholesterol**	2.009 ± 0.338	1.15 ± 0.02	11 ± 4	9 ± 1	0.45 ± 0.07
**Far-red toxin (CF®680R-BTX)-labelled nAChR in the presence of fPEG-Chol + 50 nM cholesterol**	2.127 ± 0.700	1.25 ± 0.07	27 ± 6	17 ± 4	0.53 ± 0.06
**fPEG-Chol in the presence of far-red (CF®680R-BTX) + 100 nM cholesterol**	2.163 ± 0.211	1.13 ± 0.01	9 ± 1	7 ± 1	0.50 ± 0.02
**Far-red toxin (CF®680R-BTX) in the presence of fPEG-Chol + 100 nM cholesterol**	1.451 ± 0.362	1.18 ± 0.03	40 ± 5	11 ± 1	0.71 ± 0.02
Perturbation of the submembrane actin cytoskeleton network by CK-666					
**fPEG-Chol alone upon CK-666 treatment**	1.751 ± 0.176	1.18 ± 0.01	16 ± 2	7 ± 1	0.58 ± 0.03
**fPEG-Chol in the presence of far-red (CF®680R-BTX) upon CK-666 treatment**	1.931 ± 0.100	1.11 ± 0.01	9 ± 1	8 ± 1	0.47 ± 0.02
**Far-red toxin (CF®680R-BTX)-labelled nAChR alone upon CK-666 treatment**	0.398 ± 0.064	1.13 ± 0.03	96 ± 34	16 ± 3	0.66 ± 0.03
**Far-red toxin (CF®680R-BTX)-labelled nAChR in the presence of fPEG-Chol upon CK-666 treatment**	1.117 ± 0.182	1.15 ± 0.01	31 ± 2	17 ± 3	0.61 ± 0.01

*Exogenous unlabelled cholesterol depletion/supplementation data and CK-666 treatment are included to facilitate comparisons. #Confined ratio *r* is defined as the quotient between the residence time of the trajectory in the confined state divided by the total duration of the trajectory.

In addition to the diffusion parameters obtained on the entire population of trajectories (Supplementary Table 2), Supplementary Table 3 dissects the kinetics of the trajectories in their confined and non-confined portions.

Supplementary Table 3. Generalized diffusion coefficient (K_β) and anomalous exponent (β) within confined sojourns and non-confined states of the trajectories

Experimental condition	Confined portions		Non-confined portions	
	K_β	β	K_β	β
**CF®640R-BTX**	0.483 ± 0.033	0.99 ± 0.02	2.423 ± 0.254	1.12 ± 0.05
**CF®640R-BTX upon cholesterol depletion (CDx)**	0.321 ± 0.013	0.97 ± 0.02	1.634 ± 0.151	1.10 ± 0.04
**CF®680R-BTX**	0.421 ± 0.033	1.01 ± 0.05	2.229 ± 0.265	1.09 ± 0.05
**fPEG-Chol**	1.045 ± 0.091	1.10 ± 0.01	6.446 ± 0.238	1.24 ± 0.01
**CF®680R-BTX(+fPEG-Chol)**	0.940 ± 0.119	1.06 ± 0.01	6.527 ± 0.661	1.18 ± 0.03
**fPEG-Chol(+CF®680R-BTX)**	1.407 ± 0.049	0.98 ± 0.01	7.989 ± 0.458	1.15 ± 0.01
Exogenous unlabelled cholesterol supplementation in double-labelling (toxin + fPEG-Chol) experiments				
**CF®640R-BTX (+50 nM)**	0.492 ± 0.093	1.04 ± 0.03	2.803 ± 0.436	1.29 ± 0.05
**fPEG-Chol(+CF®680R-BTX) (+50 nM)**	2.123 ± 0.998	1.12 ± 0.13	8.029 ± 1.667	1.33 ± 0.09
**CF®680R-BTX(+fPEG-Chol) (+50 nM)**	1.977 ± 0.309	1.09 ± 0.07	3.080 ± 0.727	1.28 ± 0.07
**fPEG-Chol(+CF®680R-BTX) (+100 nM)**	1.259 ± 0.188	0.96 ± 0.01	8.795 ± 1.197	1.23 ± 0.01
**CF®680R-BTX(+fPEG-Chol) (+100 nM)**	0.944 ± 0.194	1.07 ± 0.02	4.903 ± 0.868	1.25 ± 0.06

Effects of modification of the membrane cholesterol content

Table 1 in the main text lists the effects of cholesterol depletion of the membrane via CDx and cholesterol enrichment via exogenous cholesterol supplementation. Supplementary Figure 4 shows the effect of supplementation of the CHO-K1/A5 cells with aqueous-soluble cholesterol. Addition of exogenous cholesterol modified the time spent by BTX-labelled nAChRs and fPEG-cholesterol in their confined- and non-confined states.

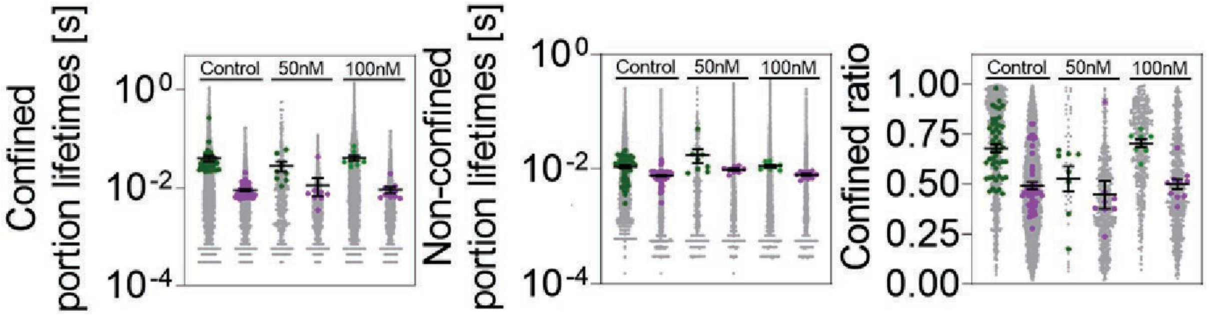

Supplementary Figure 4. Confinement parameters of the cholesterol probe and toxin-labelled nAChR trajectories. Total residence time in confinement (*left*) and non-confinement (*middle*) states and confinement ratio defined as the quotient between the residence time of the trajectory in the confined state and the total duration of the trajectory. (*right*) without (Control) and with addition of exogenous unlabelled cholesterol (concentration as labelled) and for: experimental conditions of CF®680R-BTX(+fPEG-Chol) (*green*) and fPEG-Chol(+CF®680R-BTX) (*violet*). Boxplots show the mean and SEM of the depicted parameters.

Additional confinement sojourn parameters were measured, as shown in Supplementary Figure 5. CF®640R-BTX and cholesterol-depleted (CDx) samples exhibited statistical differences in the number of steps within and between confinement sojourns ($p<0.0001$). The same was observed between fPEG-Chol alone and the cholesterol probe in the presence of BTX (fPEG-Chol(+CF®680R-BTX)) ($p<0.0001$). These differences suggest that modifying the cholesterol content of the membrane changes trajectory parameters of the BTX-labelled receptors, and vice versa.

The ratio between the residence time and trajectory duration, $r_{confined}$, was the same for CF®640R-BTX and CDx-treated samples (0.75 ± 0.01), i.e., 3/4 of the time these trajectories were confined. For CF®680R-BTX, $r_{confined}$ was 0.62 ± 0.01. Upon supplementation of cholesterol, this ratio decreased to 0.50 ± 0.01 ($p<0.0001$). In the case of fPEG-Chol, we found that in the absence and presence of CF®680R-BTX the ratio was 0.66 ± 0.01 and 0.50 ± 0.01, respectively. The confinement zone spatial dimensions, in all cases, except for fPEG-Chol, the average area distended was ~5,000 nm^2. In the case of fPEG-Chol, the surface was slightly smaller (~4,000 nm^2). In all cases, the mean major-axis length was ~50 nm.

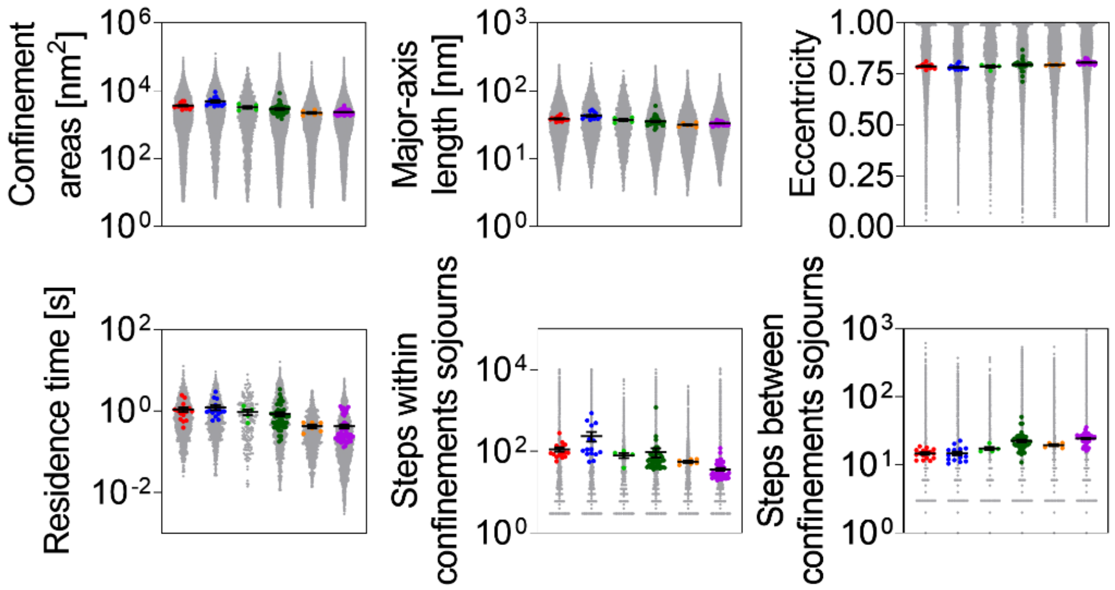

Supplementary Figure 5. Confinement sojourn parameters. Boxplots of the areas, major-axis length, eccentricities, residence times, and steps between/within confinement zones upon fitting to an ellipse. Control CF®640R-BTX (red), cholesterol-depleted samples (CDx, blue), fPEG-Chol (orange), control far-red CF®680R-BTX (green), CF®680R-BTX(+fPEG-Chol) (pink), fPEG-Chol(+CF®680R-BTX) (black).

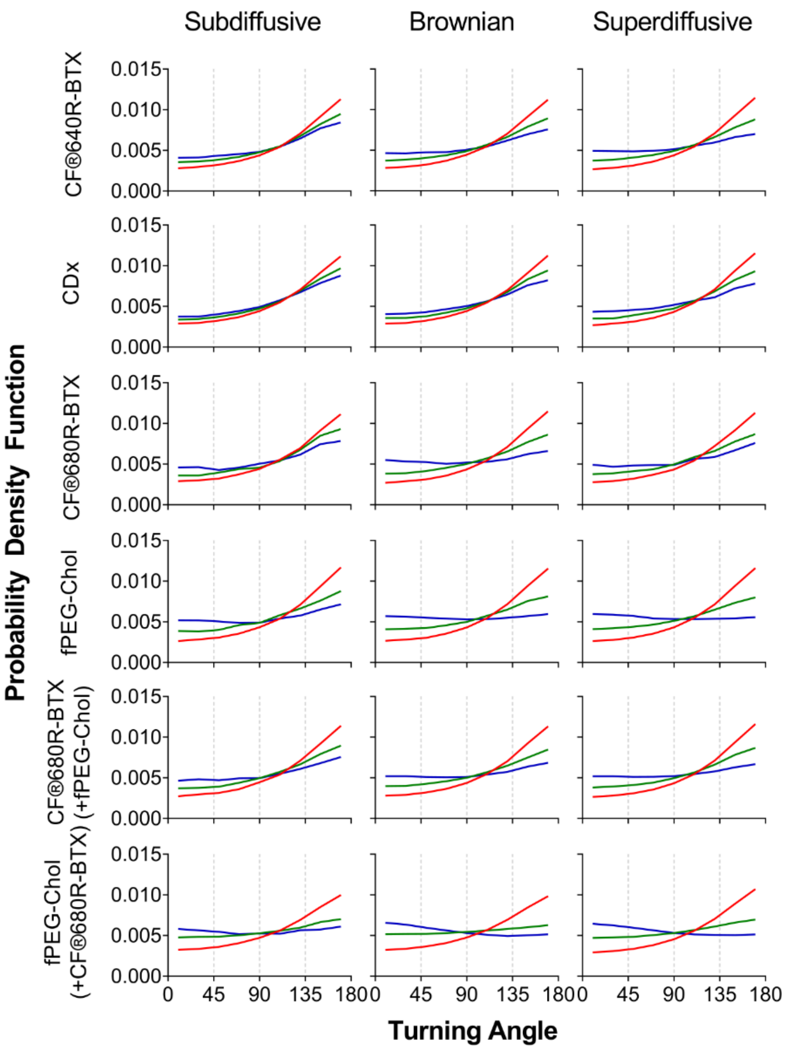

Supplementary Figure 6. **Probability density function of the turning angles across different anomalous diffusion behaviours on trajectory data upon application of the Golam and Sherman immobile-exclusion criterion (Golan and Sherman 2017)**. Trajectories were classified between diffusion regimes subdiffusive ($\beta<0.9$), Brownian ($0.9<\beta\leq1.1$) and superdiffusive ($1.1<\beta$). CF®640R-BTX, cholesterol-depleted samples (CDx), fPEG-Chol, control CF®680R-BTX, CF®680R-BTX(+fPEG-Chol), and fPEG-Chol(+CF®680R-BTX). The probability density functions (PDF) correspond to 1 (*red*), 4 (*green*), 8 (*blue*), ~~25 (*orange*), 50 (*black*)~~ step lags.

Perturbation of the actin subcortical meshwork

The elementary metrics of the trajectories upon treatment of the cells with CK-666 are listed in Supplementary Table 4 below, which lists the dynamic parameters obtained by MSD fitting according to different diffusion models (as described in Material and Methods).

Supplementary Table 4. SMT elementary parameters of nAChR and fluorescent cholesterol trajectories upon actin perturbation with CK-666

Experimental condition	Mean ± S.E.M			
	L' [number of steps]	T [track duration, s]	$\Delta_{t_i'}$[step duration, μs]	$\Delta_{x_i'}$[displacement, nm]
fPEG-Chol upon CK-666 treatment (n=1,858)	1418 ± 49	0.472 ± 0.016	333.47 ± 0.33	15 ± 1
fPEG-Chol in the presence of far-red (CF®680R-BTX) upon CK-666 treatment (n=5,927)	1169 ± 19	0.411 ± 0.007	351.71 ± 0.18	16 ± 1
Far-red toxin (CF®680R-BTX) upon CK-666 treatment (n=726)	473 ± 25	0.461 ± 0.024	974.88 ± 2.00	16 ± 1
Far-red toxin (CF®680R-BTX) in the presence of fPEG-Chol upon CK-666 treatment (n=1,261)	850 ± 34	0.623 ± 0.024	734.59 ± 1.08	15 ± 1

Distinction between confinement by physical barriers and trapped states of SMT confined sojourns

Trapping diffusion describes a particle moving with diffusion coefficient D on the plane of the membrane until it is "trapped" (i.e., immobilized) and eventually untrapped (i.e., recovers mobility) with probabilities p_{trap} and p_{untrap}, respectively. In contrast, hop diffusion describes motion of molecules within confinement sojourns imposed by physical compartments of characteristic size, like actin fences or "corrals" (Fujiwara et al. 2016; Fujiwara et al. 2023). Molecules transiently confined within the compartment boundaries can eventually move or "hop" into another compartment with probability p_{hop} (Fujiwara et al. 2016). The two restricted motions can be described by the algorithm of (Sikora et al. 2017), which defines confinement in terms of the repeated passage of a particle through the same coordinates.

The percentage of hop motion of toxin-tagged nAChR and fPEG-Chol analysed according to Eggeling and coworkers (Reina et al. 2022) and Hell and coworkers (Rickert et al. 2024) amounted to only 10-20%. We undertook an additional analysis of the motional behaviour of fluorescently-labelled molecules in the confined sojourns to further elucidate the nature of the confinement phenomenon. To this end we developed a machine learning procedure that builds on the feature-based neural network termed CONDOR (Gentili and Volpe 2021), with the specific task of discriminating between the two possible confinement mechanisms. The original CONDOR takes as input a feature vector including variables extracted from the trajectory. Here, we fed the feature vector of each confinement portion detected by the recurrent algorithm (Sikora et al. 2017) and classified the portions between the two confinement mechanisms. We also modified CONDOR to include as input the feature vector generated from the confined portions including the mean, median, standard deviation, skewness, kurtosis, and squared mean of the displacements. Step lag values between 1 and 9 steps were considered. The resulting input vector included 60 features, much less than the number in the original CONDOR. The rationale behind this choice is that the task to be accomplished here is much simpler than classifying trajectories into five theoretical models (Gentili and Volpe 2021).

We generated a dataset of 70,000 confined portions belonging to compartmentalized or trapped states, with $D \in [0.001,1]$ µm^2 s^{-1}. Next, the dataset was randomly split into validation (14,000 portions) and training (56,000 portions) datasets. To simulate compartment-limited confinement vs. trapping, a ROI of 5 µm x 5 µm was partitioned into Voronoi regions (compartments) randomly distributed across the ROI (Rickert et al. 2024). The edges of the Voronoi regions had lengths between 10 and 250 nm. Next, a particle was placed in the middle of the ROI and moved stochastically until it hit an edge of the compartment. As p_{hop} was assigned a value equal to 0, the particle bounced (Pinkwart et al. 2019). In the case of trapping, immobile particles were simulated. A Gaussian noise of 10 nm was added to all simulated confined portions. Supplementary Figure 7a shows the temporal evolution of the validation and training error as the number of epochs increased. Supplementary Figure 7b shows the corresponding confusion matrix depicting how well the trained model classified the confined portions in the validation dataset. The loss chosen for the training was binary categorical cross entropy, and Supplementary Table 5 the diffusion parameters in confinement derived from the Condor analysis.

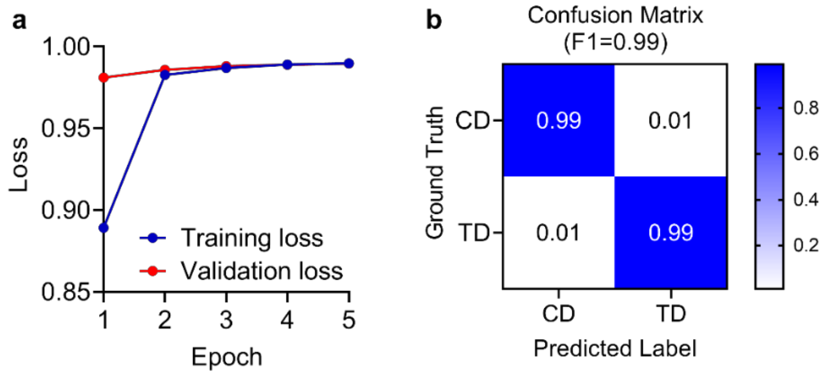

Supplementary Figure 7. CONDOR training parameters. a) The binary categorical cross entropy loss from the training and validation dataset vs. the number of epochs iterated during training. b) The confusion matrix depicts the classification performance of the trained model between compartment-driven (CD) and trapped-driven (TD) confinement discriminated across 14,000 simulated confined portions.

Supplementary Table 5. Diffusional behaviour of molecules within confinement sojourns resulting from CONDOR inference machine learning analysis (Gentili and Volpe 2021).

Condition	Compartmentalized		Trapping	
	%	Area (μm^2)	%	Area (μm^2)
**BTX640R**	93.33%	0.008 ± 0.001	6.67%	0.007 ± 0.001
**BTX680R**	94.26%	0.008 ± 0.001	5.74%	0.004 ± 0.001
**BTX680(+fPEG-Chol)**	90.12%	0.008 ± 0.001	9.88%	0.008 ± 0.001
**BTX640R upon CDx Chol depletion**	94.22%	0.010 ± 0.001	5.78%	0.012 ± 0.001
**BTX680(+fPEG-Chol) (+100 nM exogenous Chol)**	92.94%	0.007 ± 0.001	7.06%	0.003 ± 0.001
**fPEG-Chol**	88.53%	0.006 ± 0.001	11.47%	0.002 ± 0.001
**fPEG-Chol (+BTX680)**	94.58%	0.006 ± 0.001	5.42%	0.002 ± 0.001
**fPEG-Chol(+BTX680R) (50 nM exogenous Chol)**	96.94%	0.006 ± 0.001	3.06%	0.002 ± 0.001
**fPEG-Chol(+BTX680R) (+100 nM exogenous Chol)**	96.28%	0.006 ± 0.001	3.72%	0.002 ± 0.001
**fPEG-Chol upon CK-666 treatment**	91.85%	0.006 ± 0.001	8.15%	0.002 ± 0.001
**fPEG-Chol (+BTX680R) upon CK-666 treatment**	96.02%	0.006 ± 0.001	3.98%	0.002 ± 0.001
**BTX680R(+fPEG-Chol) (+ 50 nM exogenous Chol)**	89.45%	0.005 ± 0.001	10.55%	0.003 ± 0.001
**BTX680R (upon CK-666 treatment)**	96.66%	0.008 ± 0.001	3.34%	0.005 ± 0.001
**BTX680R(+fPEG-Chol) (upon CK-666 treatment)**	93.42%	0.007 ± 0.001	6.58%	0.003 ± 0.001

As shown in Supplementary Table 5, the confinement sojourns of both nAChR and fPEG-Chol molecules were largely dominated by the compartment-driven mechanism over trapping under all conditions, extending over areas of ~0.008 μm^2 and ~0.006 μm^2, respectively. fPEG-Chol compartmentalization was lower than that of the toxin-labelled receptor and slightly increased upon CK-666 treatment, while fPEG-Chol trajectories exhibited the highest, albeit small, degree of trapping among all conditions examined. Only slight changes in this behaviour were observed upon modifying cholesterol content or disrupting the submembrane actin cytoskeleton compartments by CK-666. CONDOR indicates that particles almost never remain (transiently) immobile.

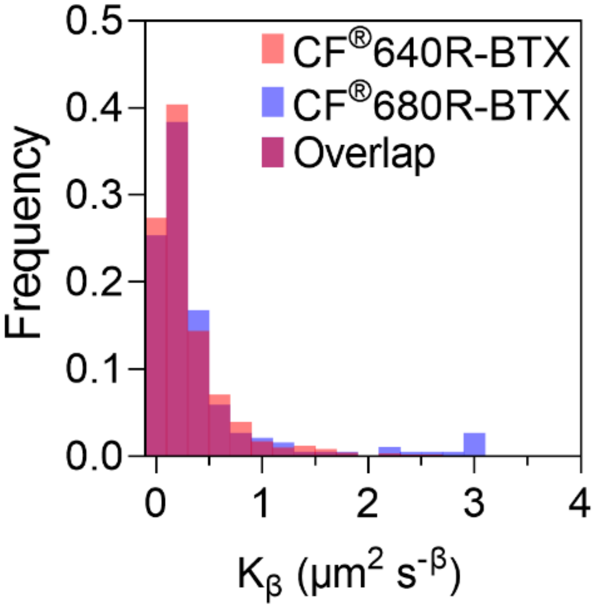

Supplementary Figure 8. Distribution of K_β values of CF®640R-BTX and CF®680R-BTX. The general diffusion coefficient of the two fluorescent toxins shows essentially the same distribution, except for the outlier superdiffusive trajectories of CF®680R-BTX, which represent only ~ 1% of the total.

Dismissal of possible biasing effects resulting from laser illumination

Fluorescence experiments are generally exposed to biasing effects from the laser illumination. Main possible light-induced effects are photobleaching, photoblinking or photoblueing of the labels (leading to loss of signal or arise of unwanted more blueish emission), phototoxic effects on cells (leading to cellular changes and death), photothermal effects (eventually heating up the membrane and thus speeding up diffusion), and laser-based trapping of objects (leading to artificial trapping of molecules. The conditions to produce such effects with deleterious changes in membrane fluidity and protein dynamics are not met by the present experimental conditions using Minflux in single-particle tracking mode, which is particularly advantageous in this regard, since: 1) focal laser intensities are one or even more orders of magnitude below those used in confocal or STED microscopy measurements, reducing detrimental effects by photobleaching, photoblinking and photo-blueing (Eggeling et al. 1998; Eggeling et al. 2005; Dasgupta et al. 2024); 2) only one excitation wavelength was used in the two-colour excitation, excluding possibilities of multi-step absorption events (Eggeling et al. 2006; Ringemann et al. 2008); 3) focal laser intensities two or more orders of magnitude higher than the ones used in the present study would be needed to produce local heating or trapping effects (Eggeling et al. 2009); 3) the red-edged excitation at ~640 nm wavelength, as used in the present work, is poorly absorbed by the cell (Mueller et al. 2011), thus minimizing phototoxic effects; and most importantly 4) prolonged exposures in the same area would be required to generate local photoinduced effects (Donnert et al. 2007; Donnert et al. 2009) – yet , it is clear that MINFLUX in single-particle tracking mode as used in the present work, definitely does not meet this condition, since the patterned doughnut-shaped beam is constantly moved to illuminate around the estimated emitter position, and rapidly refocused and zoomed in onto the moving molecule to “corral” and “lock” it to follow its trajectory, as outlined in the Introduction section of the manuscript.

Supplementary Table 6. MINFLUX scanning parameters for 2D tracking of mobile molecules

2D tracking	1st iteration	2nd iteration	3rd iteration	4th iteration
Width of the hexagon (nm)*	284	302	150	100
Pattern shape	hexagon	hexagon	hexagon	hexagon
Collected photons (counts)	40	20	20	20
Laser power multiplier (fold)	1	1	2	3
Pattern dwell time (μs)	100	100	100	100
Pattern repeat (times)	1	1	1	1
CFR	-1.0	-1.0	0.8	-1
Background threshold (kHz)	15	30	30	50

*Sometimes referred to as "*L*", the diameter of the interrogating MINFLUX bean is here called "width of the hexagon" to avoid confusion.

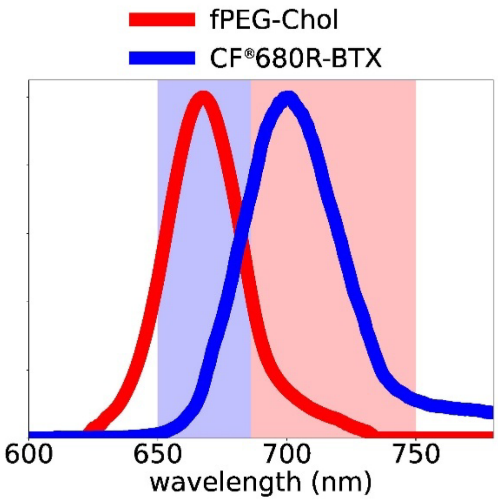

Supplementary Figure 9. Schematic depiction of the detector channel ratio (DCR) discrimination in co-labelling experiments. fPEG-Chol had an excitation maximum of 638 nm and an emission maximum of 655 nm (red curve); CF®680R-BTX and emission maximum was at 701 nm (blue curve). The DCR is defined as $I_1/(I_1+I_2)$, where I_1 and I_2 are the intensities recorded at two wavelength-distinct detection channels (fPEG-Chol channel: 650-685 nm (blue area; CF®680R-BTX channel: 685-750 nm, pink area), and represented as the effective counts at offset (ECO, referring to the photon counts collected at the outer points of the MINFLUX scanning pattern corrected for background contributions). See also Supplementary Figure 2 for additional description. Source data are provided as a Source Data file.

Supplementary Table 7. Boundaries for initializing free parameters in the optimization process.

Optimization parameters		
**Parameters**	**Lower Bound**	**Upper Bound**
D (Eq. 12)	100 nm^2 s^{-1}	100,000 nm^2 s^{-1}
σ (Eq. 12, 13, 14)	10 nm	100 nm
D_M (Eq. 13)	100 nm^2 s^{-1}	100,000 nm^2 s^{-1}
D_μ (Eq. 13)	100 nm^2 s^{-1}	100,000 nm^2 s^{-1}
L_{hop} (Eq. 13)	10 nm	1000 nm
$D_{\mu,\mathrm{conf}}$ (Eq. 14)	100 nm^2 s^{-1}	100,000 nm^2 s^{-1}
L (Eq. 14)	10 nm	1000 nm

Supplementary References

Dasgupta, A., A. Koerfer, B. Kokot, I. Urbančič, C. Eggeling, and P. Carravilla. 2024. Effects and avoidance of photoconversion-induced artifacts in confocal and STED microscopy. *Nature Methods*.

Donnert, G., C. Eggeling, and S. W. Hell. 2007. Major signal increase in fluorescence microscopy through dark-state relaxation. *Nat.Methods* 4 (1):81-86.

Donnert, G., C. Eggeling, and S. W. Hell. 2009. Triplet-relaxation microscopy with bunched pulsed excitation. *Photochem Photobiol Sci* 8 (4):481-485.

Eggeling, C., C. Ringemann, R. Medda, G. Schwarzmann, K. Sandhoff, S. Polyakova, V. N. Belov, B. Hein, C. von Middendorff, A. Schonle, and S. W. Hell. 2009. Direct observation of the nanoscale dynamics of membrane lipids in a living cell. *Nature* 457 (7233):1159-1162.

Eggeling, C., A. Volkmer, and C. A. Seidel. 2005. Molecular photobleaching kinetics of Rhodamine 6G by one- and two-photon induced confocal fluorescence microscopy. *Chemphyschem* 6 (5):791-804.

Eggeling, C., J. Widengren, L. Brand, J. Schaffer, S. Felekyan, and C. A. Seidel. 2006. Analysis of photobleaching in single-molecule multicolor excitation and Forster resonance energy transfer measurements. *J Phys.Chem.A Mol.Spectrosc.Kinet.Environ.Gen.Theory.* 110 (9):2979-2995.

Eggeling, C., J. Widengren, R. Rigler, and C. A. M. Seidel. 1998. Photobleaching of fluorescent dyes under conditions used for single-molecule detection: evidence of two-step photolysis. *Anal.Chem.* 70 (13):2651-2659.

Fujiwara, T. K., K. Iwasawa, Z. Kalay, T. A. Tsunoyama, Y. Watanabe, Y. M. Umemura, H. Murakoshi, K. G. Suzuki, Y. L. Nemoto, N. Morone, and A. Kusumi. 2016. Confined diffusion of transmembrane proteins and lipids induced by the same actin meshwork lining the plasma membrane. *Mol Biol Cell* 27 (7):1101-1119.

Fujiwara, T. K., S. Takeuchi, Z. Kalay, Y. Nagai, T. A. Tsunoyama, T. Kalkbrenner, K. Iwasawa, K. P. Ritchie, K. G. N. Suzuki, and A. Kusumi. 2023. Development of ultrafast camera-based single fluorescent-molecule imaging for cell biology. *Journal of Cell Biology* 222 (8).

Gentili, A., and G. Volpe. 2021. Characterization of anomalous diffusion classical statistics powered by deep learning (CONDOR). *Journal of Physics A: Mathematical and Theoretical* 54:314003.

Golan, Y., and E. Sherman. 2017. Resolving mixed mechanisms of protein subdiffusion at the T cell plasma membrane. *Nat Commun* 8:15851.

Kamerbeek, C. B., V. Borroni, M. F. Pediconi, S. B. Sato, T. Kobayashi, and F. J. Barrantes. 2013. Antibody-induced acetylcholine receptor clusters inhabit liquid-ordered and liquid-disordered domains. *Biophys J* 105 (7):1601-1611.

Mueller, V., C. Ringemann, A. Honigmann, G. Schwarzmann, R. Medda, M. Leutenegger, S. Polyakova, V. N. Belov, S. W. Hell, and C. Eggeling. 2011. STED nanoscopy reveals molecular details of cholesterol- and cytoskeleton-modulated lipid interactions in living cells. *Biophys J* 101 (7):1651-1660.

Pinkwart, K., F. Schneider, M. Lukoseviciute, T. Sauka-Spengler, E. Lyman, C. Eggeling, and E. Sezgin. 2019. Nanoscale dynamics of cholesterol in the cell membrane. *J Biol Chem* 294 (34):12599-12609.

Reina, F., C. Eggeling, and B. C. Lagerholm. 2022. Quantification of live cell membrane compartmentalization with high-speed single lipid tracking through interferometric Scattering Microscopy. *bioRxiv*:2021.2008.2006.455401.

Rickert, J. D., M. O. Held, J. Engelhardt, and S. W. Hell. 2024. 4Pi MINFLUX arrangement maximizes spatio-temporal localization precision of fluorescence emitter. *Proceedings of the National Academy of Sciences* 121 (11):e2318870121.

Ringemann, C., A. Schonle, A. Giske, C. von Middendorff, S. W. Hell, and C. Eggeling. 2008. Enhancing fluorescence brightness: effect of reverse intersystem crossing studied by fluorescence fluctuation spectroscopy. *Chemphyschem.* 9 (4):612-624.

Roccamo, A. M., M. F. Pediconi, E. Aztiria, L. Zanello, A. Wolstenholme, and F. J. Barrantes. 1999. Cells defective in sphingolipids biosynthesis express low amounts of muscle nicotinic acetylcholine receptor. *Eur J Neurosci* 11 (5):1615-1623.

Sato, S. B., K. Ishii, A. Makino, K. Iwabuchi, A. Yamaji-Hasegawa, Y. Senoh, I. Nagaoka, H. Sakuraba, and T. Kobayashi. 2004. Distribution and transport of cholesterol-rich membrane domains monitored by a membrane-impermeant fluorescent polyethylene glycol-derivatized cholesterol. *J. Biol. Chem.* 279 (22):23790-23796.

Sikora, G., K. Burnecki, and A. Wylomanska. 2017. Mean-squared-displacement statistical test for fractional Brownian motion. *Phys Rev E* 95 (3-1):032110.
