# Supplementary material for: Concurrent diffusion of nicotinic acetylcholine receptors and fluorescent cholesterol disclosed by two-colour sub-millisecond MINFLUX-based single-molecule tracking
Source: Nat Commun. 2025 Jul 9;16:6336. doi: 10.1038/s41467-025-61489-4 (PMC12241495; doi:10.1038/s41467-025-61489-4)
Supplement: Supplementary file 2 — Reporting summary [file 41467_2025_61489_MOESM2_ESM.pdf]

nature portfolio

Corresponding author(s): Christian Eggeling

Last updated by author(s): May 30, 2025

Reporting Summary

Nature Portfolio wishes to improve the reproducibility of the work that we publish. This form provides structure for consistency and transparency in reporting. For further information on Nature Portfolio policies, see our Editorial Policies and the Editorial Policy Checklist.

Statistics

For all statistical analyses, confirm that the following items are present in the figure legend, table legend, main text, or Methods section.

n/a	Confirmed	
☐	☒	The exact sample size (*n*) for each experimental group/condition, given as a discrete number and unit of measurement
☐	☒	A statement on whether measurements were taken from distinct samples or whether the same sample was measured repeatedly
☐	☒	The statistical test(s) used AND whether they are one- or two-sided<br>*Only common tests should be described solely by name; describe more complex techniques in the Methods section.*
☐	☒	A description of all covariates tested
☐	☒	A description of any assumptions or corrections, such as tests of normality and adjustment for multiple comparisons
☐	☒	A full description of the statistical parameters including central tendency (e.g. means) or other basic estimates (e.g. regression coefficient) AND variation (e.g. standard deviation) or associated estimates of uncertainty (e.g. confidence intervals)
☐	☒	For null hypothesis testing, the test statistic (e.g. *F*, *t*, *r*) with confidence intervals, effect sizes, degrees of freedom and *P* value noted<br>*Give P values as exact values whenever suitable.*
☐	☒	For Bayesian analysis, information on the choice of priors and Markov chain Monte Carlo settings
☒	☐	For hierarchical and complex designs, identification of the appropriate level for tests and full reporting of outcomes
☒	☐	Estimates of effect sizes (e.g. Cohen's *d*, Pearson's *r*), indicating how they were calculated

Our web collection on statistics for biologists contains articles on many of the points above.

Software and code

Policy information about availability of computer code

Data collection	No code has been used to collect data.
Data analysis	Python code for the analysis of the data is available at the public repository https://github.com/lucasSaavedra123/minflux_analysis.

For manuscripts utilizing custom algorithms or software that are central to the research but not yet described in published literature, software must be made available to editors and reviewers. We strongly encourage code deposition in a community repository (e.g. GitHub). See the Nature Portfolio guidelines for submitting code & software for further information.

Data

Policy information about availability of data

All manuscripts must include a data availability statement. This statement should provide the following information, where applicable:

- Accession codes, unique identifiers, or web links for publicly available datasets
- A description of any restrictions on data availability
- For clinical datasets or third party data, please ensure that the statement adheres to our policy

All data gathered for this study is available upon request to the corresponding authors. Upload of the data on a public data server has so far not been conducted due to the mere amount of data.

Research involving human participants, their data, or biological material

Policy information about studies with human participants or human data. See also policy information about sex, gender (identity/presentation), and sexual orientation and race, ethnicity and racism.

Reporting on sex and gender	No gender-specific material or data has been used in this study.
Reporting on race, ethnicity, or other socially relevant groupings	No data relevant to race, ethnicity, or other socially relevant groupings has been employed n this study.
Population characteristics	Not relevant - see above.
Recruitment	Not relevant - see above.
Ethics oversight	*Identify the organization(s) that approved the study protocol.*

Note that full information on the approval of the study protocol must also be provided in the manuscript.

Field-specific reporting

Please select the one below that is the best fit for your research. If you are not sure, read the appropriate sections before making your selection.

☒ Life sciences ☐ Behavioural & social sciences ☐ Ecological, evolutionary & environmental sciences

For a reference copy of the document with all sections, see nature.com/documents/nr-reporting-summary-flat.pdf

Life sciences study design

All studies must disclose on these points even when the disclosure is negative.

Sample size	Following our experience in single-particle tracking we extracted parameters from sample sizes n > 1000 only.
Data exclusions	No data has been excluded.
Replication	Data has been reproduced on different measurements days and sample preparations.
Randomization	Allocation into different experimental groups has been performed according to sample treatments like labeling, cholesterol or actin treatment.
Blinding	Non blinding has been performed.

Reporting for specific materials, systems and methods

We require information from authors about some types of materials, experimental systems and methods used in many studies. Here, indicate whether each material, system or method listed is relevant to your study. If you are not sure if a list item applies to your research, read the appropriate section before selecting a response.

Materials & experimental systems

n/a	Involved in the study	
☒	☐	Antibodies
☐	☒	Eukaryotic cell lines
☒	☐	Palaeontology and archaeology
☒	☐	Animals and other organisms
☒	☐	Clinical data
☒	☐	Dual use research of concern
☒	☐	Plants

Methods

n/a	Involved in the study	
☒	☐	ChIP-seq
☒	☐	Flow cytometry
☒	☐	MRI-based neuroimaging

Eukaryotic cell lines

Policy information about cell lines and Sex and Gender in Research

Cell line source(s)	The CHO-K1/A5 cells were generated in the laboratory of the author Francisco Barrantes, as detailed in (Roccamo et al.

Cell line source(s)	1999).
Authentication	No authentication used.
Mycoplasma contamination	All cell lines were tested for negative mycoplasma contamination.
Commonly misidentified lines (See ICLAC register)	Not relevant

Plants

Seed stocks	Not relevant
Novel plant genotypes	Not relevant
Authentication	Not relevant
